# Effects of school-based high-intensity interval training on body composition, cardiorespiratory fitness and cardiometabolic markers in adolescent boys with obesity: a randomized controlled trial

**DOI:** 10.1186/s12887-021-03079-z

**Published:** 2022-03-01

**Authors:** Cao Meng, Tang Yucheng, Li Shu, Zou Yu

**Affiliations:** 1 Institute of Physical Education, Normal College, Shenzhen University, 3688 Nan Hai Road, Nan Shan district, Shenzhen, 518061 China; 2 Institute of KEEP Collaborative Innovation, Shenzhen, 518061 China; 3 College of Education, Zhejiang University, Zhejiang, 310058 China

## Abstract

**Background:**

With accumulating evidence suggesting that CVD has its origins in childhood obesity. The purpose of this study was to determine the effect of a real-world school-based high-intensity interval training intervention on body composition, cardiorespiratory fitness and cardiometabolic markers in obese boys aged 10 to 13 years.

**Methods:**

Forty-five adolescent boys with obesity (age = 11.2 ± 0.7 years, BMI = 24.2 ± 1.0 kg/m^2^), were randomized to high-intensity interval training group (HIIT, *n* = 15), moderate-intensity continuous training group (MICT, *n* = 15), or a control group (CON, *n* = 15). The intervention groups performed three weekly exercise sessions over 12 weeks. HIIT group performed two sets of eight bouts of 15 s run at high-intensity [90 ~ 100% maximal aerobic speed (MAS)] separated by eight bouts of 15 s recovery run at low-intensity (50% MAS), MICT group performed 30 min run at moderate intensity (60 ~ 70% MAS) and CON group were instructed to continue their normal behaviors. All participants had indices of body composition, cardiorespiratory fitness (CRF) and cardiometabolic markers measured at baseline and post-intervention. Statistical differences between and within groups were determined by use of two-way analysis of variance (ANOVA) with repeated measures.

**Results:**

Following the school-based training program, BMI and body fat mass decreased (BMI: − 1.8 kg/m^2^ vs. – 1.2 kg/m^2^, *P* < 0.01; FM: − 1.6 kg, *P* < 0.05 vs. -3.7 kg, *P* < 0.01) in HIIT and MICT group, but there was no significant difference between the two interventions; $\dot{\mathrm{V}}{\mathrm{O}}_{2\mathrm{peak}}$ both increased significantly in two intervention groups, and the increment of HIIT group was significantly greater than that of MICT (6.1 mL/kg/min vs. 3.8 mL/kg/min, *P* < 0.01), Visceral adipose tissue was significant decrease in HIIT group (− 53 g vs. -17 g, *P* < 0.01) whilst the MICT group experienced a significant decrease in body fat percentage (− 3.1 ± 1.0 kg, *P* < 0.01), but there were no significant difference between the two interventions. Low-density lipoprotein cholesterol decreased only in HIIT group (− 17.2%, *P* < 0.05). Significant decrease in the usual index of insulin resistance (HOMA-IR) occurred in HIIT and MICT groups (− 27.3 and − 28.6%, respectively; *P* < 0.05).

**Conclusions:**

Our results demonstrated that high-intensity interval training based on running can be used to improve the physical health of obese adolescents in school. Further investigations involving a larger cohort of participants, taken from different schools, is recommended.

**Trial registration:**

*title* Effect of High Intensity Interval Training on Obese Children and Adolescents, *time* 16/12/2017, *ID* ChiCTR-IOR-17013992, *website* http://www.chictr.org.cn

## Background

Childhood obesity is one of the most serious health challenges of the twenty-first century [1]. Compared to 1980, the worldwide prevalence of adolescents classified as obese in 2013 from developed countries increased from 16.2 to 22.6%. In China, the national survey in 2018 showed that the obesity rate of adolescents reached 16.0% [2]. Since adolescents classified as obese have higher morbidity and mortality risks compared to normal weight counterparts and are of higher risk of becoming adults with obesity, pediatric obesity is of foremost public health concern [3]. Physical activity is one of the most important interventions to reduce adolescent obesity, WHO recommends that children and adolescents should engage in an average of 60 min of moderate to high intensity physical activity (MVPA) per day to obtain health benefits [4]; however, more than 80% of adolescents fail to reach the minimum recommended amount of physical activity [2]. Given that adolescents report difficulty starting and adhering to traditional exercise prescription, there is a need to explore and develop engaging alternatives for youth to achieve the many health benefits related to regular physical activity.

Exercise is a critical component in the management of pediatric obesity. Traditionally, moderate-intensity continuous training (MICT) has been the most common type of exercise recommended to improve body composition and cardiorespiratory fitness (CRF) [5, 6]. In recent years, growing evidence under laboratory conditions showed that HIIT is more time-efficient than MICT in improving body composition or other health parameters of obese children and adolescents [7–10]. Enjoyment of children during and after HIIT is important, and consequent long-term exercise adherence [11]. Previous study suggested that the intensity of physical activity has been found to be negatively associated with exercise adherence in overweight children [12]. However, Malik and his colleagues suggested that affect responses during HIIT are dependent on the intensity of the work-interval and are not entirely negative (unpleasant feelings). The higher intensity HIIT protocol (85% peak power) has similar pleasure with the lower HIIT (70% peak power), and only 85% HIIT elicits sufficient HR stimulus to facilitate potential health benefits [13]. Schools are an ideal setting to implement physical activity programs targeted at youths’ learning and intellectual abilities, allow children to obtain exercise methods through PA interventions carried out in schools, allowing them to participate in PA outside of school (such as community sports) [14]. There are a few school-based HIIT intervention studies of normal weight and obese children [15–17]. The available evidence suggested that HIIT programs could be carried out in conjunction with physical education (PE) class activities or in specific periods during the school day [18]. Real-world effectiveness studies are required that trial low-cost, accessible HIIT protocols over the long-term. In this context, the use of high-intensity interval running requiring a small physical space in non-laboratory environment and more suitable for school is an appealing option.

Therefore, the aim of this study was to examine the effects of 12 weeks of school-based HIIT and MICT protocol using a field approach on body composition, CRF and cardiometabolic markers in obese boys.

## Methods

### Study design

A parallel groups design was adopted, with an exercise intervention period lasting 12 weeks. Before the intervention, the subjects were measured in three parts separated by 1 day. The first part involved blood sample collection. Second, a graded exercise testing (GXT) until exhaustion to determine peak oxygen uptake ($\dot{\mathrm{V}}{\mathrm{O}}_{2\mathrm{peak}}$), and finally, dual-energy x-ray absorptiometry (DXA) and anthropometry was carried out. The post-test was conducted three days after the training, and each subject was measured according to the pre-test order. DXA and blood sample collection were measured in the hospital, graded exercise test (GXT) was carried out in the laboratory, the measurement of other parameters and exercise intervention were completed in the school.

### Participants

A total of 45 adolescent boys (age = 11.2 ± 0.7 years, BMI = 24.2 ± 1.0) with obesity as defined by Cole et al. [19] were enrolled into this randomized controlled trial (ChiCTR-IOR-17013992) from Experimental School in China, this trial was registered on 16/12/2017. Convenience sampling was employed based on our school-based recruitment through local pediatrician consultations. To be included, participants had to: (1) be aged between 11 and 13 years; (2) be between Tanner stage 1 to 3 [20] (pubertal stage was evaluated according to the Tanner classification by a trained pediatrician); (3) present a BMI greater than or equal to the 95th percentile for their gender and age; (4) be free of any medication that could interact with the protocol (e.g., cardiac abnormalities, hypertension, diabetes, orthopedic, neuromuscular, or neurological disorders); (5) present no contraindication to physical activity; (6) self-report less than 2 h of physical activity per week (International Physical Activity Questionnaire – IPAQ). All participants and their legal representatives received information sheets and signed consent forms as requested by the local ethical authorities. After the enrollment to the study, the participants were randomized into 3 groups for 12 weeks: (1) high-intensity interval training (HIIT, *n* = 15), (2) moderate-intensity continuous training (MICT, *n* = 15), or (3) non-exercising control (CON, *n* = 15). First, allocation concealment was carried out. In this study, a simple randomization method was used to generate random numbers by SPSS software. The person who determined the random assignment table did not participate in the inclusion of subjects. The random distribution form is made in triplicate, one for each of the project leader, the school principal and the statistician. The participants and data Analyst were blinded after allocation. During the intervention, participants in different exercise intervention groups were trained respectively. The independent researcher is responsible for statistical analysis of the data to ensure that he does not know the specific distribution of interventions. The study protocol was approved by the ethics review committee at the Shanghai University of Sport (Protocol ID: 2018019–2018) in accordance with the Declaration of Helsinki.

### Anthropometry, blood pressure, and body composition measures

Body mass (BM) was measured using a TANITA scale (Tanita BC-533, Tokyo, Japan), stature was measured in centimeters without shoes, heels together, and the back of the subject parallel to the stadiometer (RGZ-120-RT, Shanghai, China). Body mass index (BMI) was calculated from BMI = BM [kg] ÷ Height^2^ [m^2^]. Waist circumference (WC; in cm) was measured with a non-deformable tape measure between the lower rib margin and the iliac crest, at the end of normal expiration.

After relaxation in a seated position for approximately over 5 min, resting systolic and diastolic blood pressures (SBP and DBP) were measured using an automatic BP monitor (Omron BP652, Omron Healthcare Inc., Vernon Hills, IL, USA).

Total body fat mass (FM, kg), body fat percentage (%BF), fat free mass (FFM, kg) and estimated visceral adipose tissue mass (VAT, g) were obtained through whole-body DXA scans (Lunar Prodigy, GE Healthcare, USA). Before the daily measurement, research team member with a teaching qualification will carry out quality and calibration test, and conduct quality control body mold correction after the machine is started. The phantom scan measurement value is consistent with its standard value (± 1%), which is regarded as a quality assurance pass. The measurement method uses a whole-body scan, and the specific operating steps follow the instrument’s instruction manual. To reduce errors, fasting measurement (10 h fast) and all DXA measurements are performed by the same trained professional. Before the measurement, subject was in a supine position and kept still during the measurement.

### Cardiorespiratory fitness and maximal aerobic speed

$\dot{\mathrm{V}}{\mathrm{O}}_{2\mathrm{peak}}$ and maximal heart rate (HR_max_) were measured using a continuous incremental exercise test to exhaustion on a treadmill and computerized metabolic system (MAX-IIa Metabolic System, AEI Technologies, USA). Children commenced the test at 4 km/h and walked at that intensity for 1 min. The treadmill speed was then slowed to a stop (0 km/h) to allow a 1 min recovery, before being increased again to a speed of 6 km/h for a further minute of exercise. A 1 min recovery was implemented following each minute of active exercise. Increments of 1 km/h continued with this protocol until a speed of 8 km/h was accomplished. Thereafter, increments in running speeds of 0.5 km/h occurred (8.5, 9.0 km/h, etc.) until volitional exhaustion. Heart rate was monitored using a heart rate monitor (Polar team Oh1, Polar, Kemele, Finland). Exhaustion was verified based on the following criteria: (1) a plateau in oxygen uptake, (2) respiratory exchange ratio ≥ 1.1, (3) peak heart rate ± b.p.m. of the predicted maximal heart rate (220 – age), and (4) apparent voluntary exhaustion. At least two of the four criteria were met or the test was repeated. In order to ensure the safety of subjects, we will stop the test immediately when heart rate close to 200 b.p.m. or they gave up voluntarily, and $\dot{\mathrm{V}}{\mathrm{O}}_{2\mathrm{peak}}$ was identified [21].

The 20-m shuttle run test (20-mSRT) was conducted two days after the treadmill GXT. Maximal aerobic speed (MAS) was measured using the 20-mSRT as previously documented [22, 23]. The 20-mSRT has been validated as a predictor of maximal aerobic capacity in young people [24] and is familiar with all youth. Participants will be instructed to run between two lines separated by 20-m, while keeping pace with the audio signals emitted from a MP3 produced by the National Coaching Foundation. The test was terminated when the participant could no longer complete the 20 m run within the allotted time on two consecutive attempts. The speed at the last completed stage was considered as the MAS (km/h).

### Fasting blood samples

Fasting blood draws (10 h fast) were performed at least 48 h after the final exercise session for the determination of glucose, insulin and lipids. Blood samples for glucose and lipids were processed in duplicate by a local Pediatric hospital. Total cholesterol (TC), high-density cholesterol (HDL) and triglyceride (TG) levels were measured by enzymatic methods, and low-density cholesterol (LDL) concentrations were derived using the Frielwald et al. formula. Blood glucose concentrations were measured using an automated device (AU2700, Olympus, France). Blood insulin was assayed by an IRMA Insulin kit (Immunotech, France). Insulin resistance was assessed using the homeostatic model assessment for insulin resistance (HOMA-IR) which has been computed as follows:

HOMA-IR = [Fasting insulin (μU/ml)] × Fasting glucose (mmol/L)]/22.5.

### Training interventions

Exercise training with HIIT or MICT was 3 separate days per week (e.g., Monday, Wednesday and Friday) for 12 weeks. All training sessions were carried out on an outdoor track. Training sessions were always preceded by a 5-min warm-up and cool-down at 55 ~ 60% HR_max_. The warm-up session included moderate-intensity jogging (3 min), dynamic stretching (1 min), and acceleration running (1 min).

For MICT, participants ran 30-min at 60% of MAS during the first 4 weeks, then the training intensity was increased to 65% for weeks 5–8, 70% for weeks 9–12. For HIIT protocol, participants ran at 90% of MAS for the first 4 weeks, and increased to 95% for weeks 5–8, 100% for weeks 9–12. Training data for the two training groups are outlined in Table 1. Participants were excluded from the study if they were not able to make up a missed training session within the same week.

**Table 1:** Exercise training data for the two training groups (mean ± SD)

	Weeks 1–4	Weeks 5–8	Weeks 9–12
*HIIT (n = 15)*			
Work:Rest interval duration, s	15:15	15:15	15:15
Work:Rest interval intensity	90:50% MAS	95:50% MAS	100:50% MAS
Number of repetitions	8	8	8
Number of sets	2	2	2
Rest between sets, s	180	180	180
MHR, b.p.m	163 ± 16	168 ± 13	165 ± 14
%HR_max_, %	81 ± 0	80 ± 0	81 ± 1
Duration, min	11	11	11
Attendance, %	91 ± 2	85 ± 11	91 ± 7
*MICT (n = 15)*			
Work intensity	60% MAS	65% MAS	70% MAS
MHR, b.p.m	137 ± 11	142 ± 13	141 ± 12
%HR_max_, %	71 ± 1	70 ± 1	70 ± 0
Duration, min	30	30	30
Attendance, %	84 ± 4	85 ± 12	87 ± 7

Data shown for participants who completed the intervention. *MHR* mean heart rate of training

* Training duration for HIIT includes 8 min exercise and 3 min resting interval, i.e., one 3-min breaks among 2 HIIT sets

For the HIIT group, participants were placed in different lanes of the tract according to the individual MAS, performed two sets of eight 15-s bouts of high-intensity run (90 ~ 100% MAS, about 80 ~ 90% HR_max_) separated by eight 15-s recovery bouts at low-intensity (50% MAS, about 40 ~ 60% HR_max_), 3-min rest between two sets, total duration time was 11-min. Participants maintained the correct running speed by listening to pre-recorded sounds throughout the training session. For example, a subject who had a MAS 9.0 km/h (2.5 m/s), he had to complete 37.5 m in 15-s, (i.e., 100% of MAS), which was followed by an active recovery to run over 18.8 m in 15-s, i.e., at 50% of MAS. After completion of this 15 s:15 s bout, the subject turned around and ran back to repeat the bout in the opposite way with the same intensity. Participants in the MICT group were instructed to maintain the correct running speed and distance by periodically checking speed and distance data in the watch used to record HR. All the HIIT and MICT sessions were supervised by a teacher of the research team to ensure that participants performed each training session appropriately. After 4 and 8 weeks of training, participants performed a new 20 m-SRT for the adjustment of the speed of the training program. Participants in the control group did not have any exercise training, but they maintained their normal daily routine. All activities were performed in the school setting, in activity class time, with a total of 36 sessions lasting 30–40 min each. All three groups continue to participate their regular physical education (PE) classes scheduled by the school.

We required the subjects to run the corresponding distance in 15 s (according to their MAS). In the first 4 weeks, 3 to 5 subjects (20 ~ 30%) could not reach the specified distance due to physical reasons in the final stage of training session. After 4 weeks of training, with the improvement of subjects’ cardiorespiratory fitness, even if we corrected the MAS (extended distance), almost all subjects could complete 15-s of high-intensity running per bout.

### Dietary intake

Daily energy intake was estimated with a validated 24-h dietary recalls (3 weekdays and 1 weekend day) [25], during the initial and the end of the training program were carried out by all participants with the help of their parents and/or the investigators. Energy intake based on the dietary records were calculated with a commercial software (Boohee health software, Boohee Info Technology Co., Shanghai, China), averaged and reported as kilocalories per day (kcal/day). Participants were asked to maintain their current diet throughout the duration of the study.

### Statistical analysis

The sample size calculation using G*Power 3.1 [26] was based on previously reported data regarding $\dot{\mathrm{V}}{\mathrm{O}}_{2\mathrm{peak}}$ in overweight and obese adolescents following moderate and high intensity interval exercise. Tjonna et al. [27] demonstrated a 9.2% difference in percent change of $\dot{\mathrm{V}}{\mathrm{O}}_{2\mathrm{peak}}$ between the moderate and high intensity groups from pre- to post-intervention. With a 2-sided, 0.05 significance level and $\dot{\mathrm{V}}{\mathrm{O}}_{2\mathrm{peak}}$ as the primary variable, 6 subjects in each group would allow us to detect a significant difference between exercise groups at 80% power. Data analysis was performed using the SPSS Statistical Software (v20.0; SPSS Inc., Chicago, IL, USA). Shapiro-Wilk and Levene’s tests were carried out to determine normality of the data distribution and homogeneity of variance, respectively, for all outcome measures. Nonparametric Kruskal-Wallis test was used when data was not normally distributed. All data passed the normality and homogeneity tests. A two-way analysis of variance (ANOVA) with repeated measures (HIIT vs. MICT vs. CON × 2 times: pre- vs. post-intervention). Post-hoc test (with Bonferroni) was applied if the main factor was significant. Analysis of covariance (ANCOVA) was used to assess the role of age and baseline value on the significant differences within the group and between groups. In addition, post hoc, effect size statistics (ES) for all the statistically significant t ratios were also established. These calculations were based on Cohen’s classification and knowledge of the ES enabled estimating the magnitude of the difference (i.e., trivial: ES < 0.2, small: 0.2 ≤ ES < 0.5, moderate: 0.5 ≤ ES < 0.8, and large: ES ≥ 0.8). The level of significance was set at *P* ≤ 0.05.

## Results

**Fig. 1:**
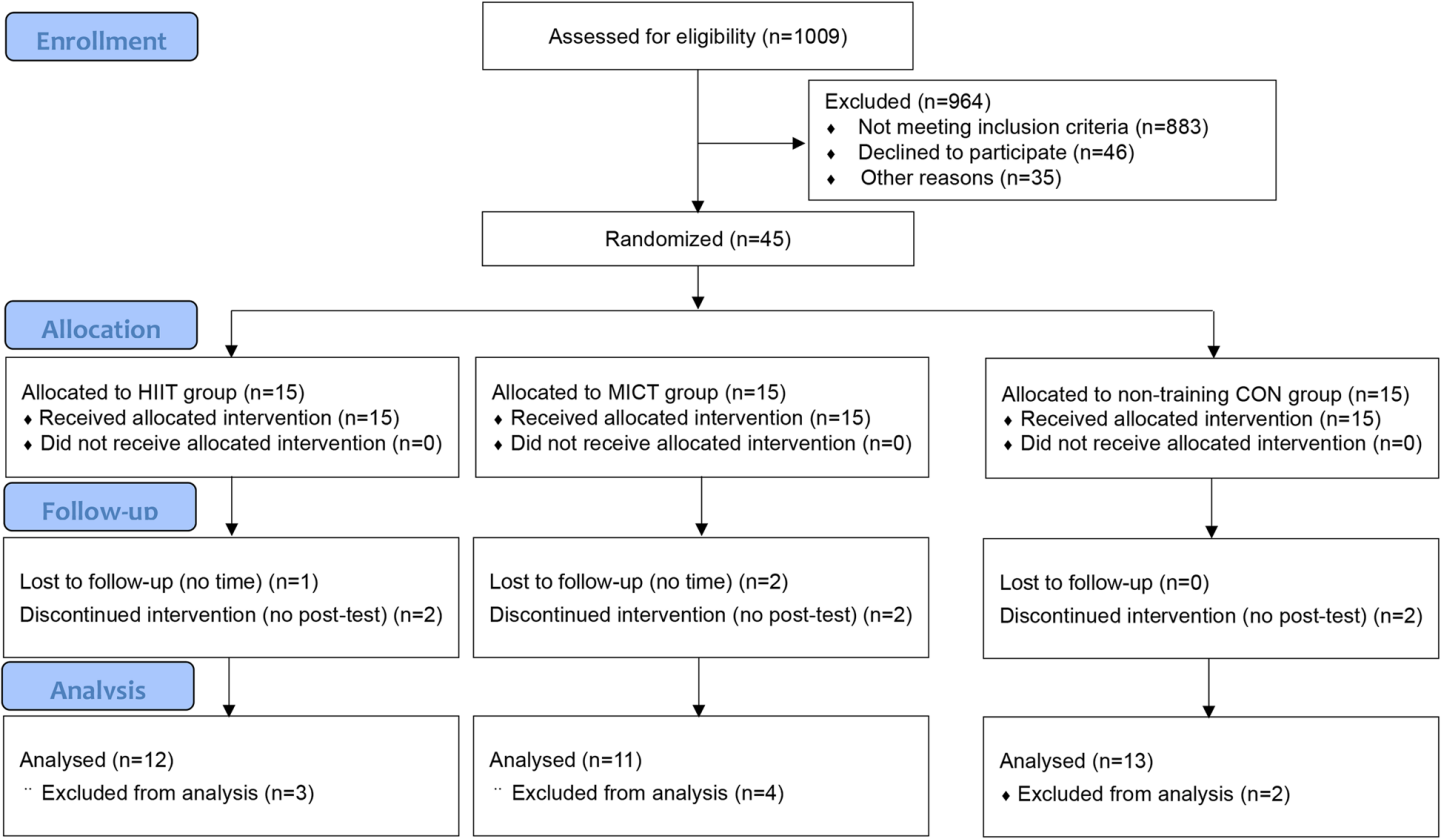
Flow chart of participant enrollment, randomized group allocation, and final analysis

Of the 126 participants who entered the run-in phase, 45 (35.7%) were randomized. The other 81 participants were not randomized because of not sign informed consent (*n* = 46) and no time (*n* = 35). During the 12-week intervention period no injuries were reported but nine participants were unable to complete the training program for personal reasons: 3 in the HIIT group, 4 in the MICT group and 2 in the CON group, and their data are thus excluded from all analyses. Therefore, 36 obese boys have fully completed the current study (Fig. 1). They had similar daily caloric intakes in three group (2937 ± 271 kcal, 3129 ± 253 kcal and 2989 ± 183 kcal, respectively, *P* > 0.05), although all of them had much higher than the recommended daily calories (1800 ~ 2600 for boys) by American Heart Association [28]. The values of all body composition, CRF and cardiometabolic markers variables and energy intake, measured before and after the intervention period, are presented in Tables 2 and 3.

**Table 2:** Baseline physical characteristics of three groups **(**mean ± SD**)**

	HIIT (*n* = 12)	MICT (*n* = 11)	CON (*n* = 13)
Age (y)	11.4 ± 0.8	11.2 ± 0.7	11.0 ± 0.7
Height (m)	148.8 ± 7.8	152.5 ± 7.9	151.0 ± 6.3
Body mass (kg)	56.5 ± 8.1	56.2 ± 10.1	55.3 ± 5.7
BMI (kg/m^2^)	24.5 ± 1.1	24.4 ± 0.9	23.8 ± 0.8
%BF (%)	38.4 ± 4.0	39.2 ± 2.7	38.3 ± 1.6
FM (kg)	21.7 ± 2.2	22.0 ± 4.6	20.5 ± 2.0
FFM (kg)	30.2 ± 3.6	30.2 ± 5.4	28.7 ± 2.5
WC (cm)	83.8 ± 7.4	79.2 ± 5.6	82.5 ± 7.1
$\dot{\mathrm{V}}{\mathrm{O}}_{2\mathrm{peak}}$ (mL/kg/min)	41.8 ± 1.7	42.1 ± 2.6	42.8 ± 1.3
SBP (mmHg)	115 ± 7	119 ± 6	115 ± 5
DBP (mmHg)	69 ± 5	71 ± 4	71 ± 4
Energy intake (Kcal/day)	2937 ± 271	3129 ± 253	2989 ± 183

*%BF* body fat percentage; *BMI* body mass index; *DBP* diastolic blood pressure; *FFM* fat free mass; *FM* total body fat mass; *SBP* systolic blood pressure; $\dot{V}{O}_{2 peak}$ peak oxygen uptake; *WC* waist circumference;

**Table 3:** Cardiometabolic and fitness variables at baseline and post-interventions (mean ± SD)

Variable	HIIT (*n* = 12)		MICT (*n* = 11)		CON (*n* = 13)		Time	Group	Time × Group
	Baseline	Post	Baseline	Post	Baseline	Post			
*Energy intake (Kcal/day)*	2937 ± 271	3061 ± 163	3129 ± 253	3105 ± 177	2989 ± 183	3052 ± 176	0.267	0.128	0.490
*Body composition*									
BMI (kg/m^2^)	24.5 ± 1.1	22.7 ± 1.0 ^#, £^	24.4 ± 0.9	23.2 ± 0.7 ^#^	23.8 ± 0.8	24.8 ± 1.0 ^*^	0.003	0.029	0.000
%BF (%)	38.4 ± 4.0	36.2 ± 3.9 ^£^	37.5 ± 2.0	34.4 ± 1.5 ^#, £^	38.3 ± 1.6	39.5 ± 2.1	0.033	0.119	0.022
FM (kg)	21.7 ± 2.2	19.3 ± 1.7 ^*, £^	22.0 ± 4.6	18.3 ± 3.4 ^#, £^	20.5 ± 2.0	23.3 ± 2.6 ^*^	0.107	0.085	0.001
FFM (kg)	30.2 ± 3.6	32.1 ± 4.5	30.2 ± 5.4	29.4 ± 4.3	28.7 ± 2.5	30.5 ± 3.0	0.327	0.335	0.450
WC (cm)	83.8 ± 7.4	78.8 ± 6.1	79.2 ± 5.6	78.5 ± 7.5	82.5 ± 7.1	84.9 ± 7.2	0.496	0.061	0.164
VAT (g)	348 ± 55	295 ± 52 ^*, £^	313 ± 86	296 ± 81 ^£^	355 ± 68	394 ± 71	0.425	0.003	0.063
*Cardiorespiratory fitness*									
$\dot{\mathrm{V}}{\mathrm{O}}_{2\mathrm{peak}}$(mL/kg/min)	41.8 ± 1.7	47.9 ± 2.6 ^#, £^	42.6 ± 1.6	45.6 ± 2.1 ^#, £^	42.8 ± 1.3	42.6 ± 2.9	0.000	0.014	0.000
SBP (mmHg)	115 ± 7	110 ± 5 ^*, £^	119 ± 6	114 ± 7 ^*^	115 ± 5	116 ± 4	0.029	0.058	0.090
DBP (mmHg)	69 ± 5	67 ± 3 ^£^	71 ± 4	68 ± 4 ^£^	71 ± 4	71 ± 3.7	0.087	0.022	0.342
*Cardiometabolic markers*									
Glucose (mmol/L)	4.9 ± 0.3	4.7 ± 0.3 ^£^	5.0 ± 0.3	4.9 ± 0.4 ^£^	5.2 ± 0.4	5.4 ± 0.4	0.709	0.000	0.146
Insulin (μIU/mL)	10.2 ± 4.9	7.5 ± 2.8 ^£^	9.6 ± 4.3	6.8 ± 3.0 ^£^	10.6 ± 3.3	12.3 ± 2.4	0.142	0.007	0.049
HOMA-IR	2.2 ± 1.1	1.6 ± 0.6 ^*, £^	2.1 ± 1.0	1.5 ± 0.6 ^*, £^	2.4 ± 0.7	2.9 ± 0.6	0.164	0.001	0.012
TC (mmol/L)	4.0 ± 0.6	4.0 ± 0.5	4.2 ± 0.8	3.9 ± 0.5	4.0 ± 0.5	4.2 ± 0.6	0.943	0.825	0.378
TG (mmol/L)	1.6 ± 0.4	1.4 ± 0.3	1.4 ± 0.7	1.3 ± 0.5	1.3 ± 0.5	1.5 ± 0.6	0.974	0.520	0.347
HDL (mmol/L)	1.3 ± 0.3	1.5 ± 0.2 ^£^	1.4 ± 0.3	1.4 ± 0.2	1.2 ± 0.2	1.3 ± 0.2	0.112	0.034	0.606
LDL (mmol/L)	2.3 ± 0.2	1.9 ± 0.3 ^*^	2.3 ± 0.4	2.0 ± 0.2	2.1 ± 0.5	2.2 ± 0.5	0.034	0.954	0.107

*%BF* body fat percentage; *BMI* body mass index; *DBP* diastolic blood pressure; *FFM* fat free mass; *FM* total body fat mass; *HDL* high-density lipoprotein cholesterol; *HOMA-IR* homeostatic model assessment for insulin resistance; *LDL* low-density lipoprotein cholesterol; *SBP* systolic blood pressure; *TC* total cholesterol; *TG* triglyceride; *VAT* visceral adipose tissue; $\dot{V}{O}_{2 peak}$ peak oxygen uptake; *WC* waist circumference;

Significantly different within each group before vs after program: ^*^ *P* < 0.05, ^#^*P* < 0.01

Significantly different from the other groups: ^§^ *P* < 0.05

Significantly different from control: ^£^ *P* < 0.05

### Body composition

The comparison of the three groups showed that their anthropometric parameters and age before training are matched. There was no significant difference in energy intake among groups.

At the post-intervention, we noted a significant decreased in the BMI and FM of HIIT group (*P* < 0.01, ES = 0.81 and *P* < 0.05, ES = 0.62) and MICT group (*P* < 0.01, ES = 0.48 and *P* < 0.01, ES = 0.92). %BF was significantly decreased in MICT (− 3.1 ± 1.0 kg, *P* < 0.01, ES = 0.49) and VAT significantly decreased in HIIT (− 53 ± 16 g, *P* < 0.05, ES = 0.34). In the between groups’ comparison, %BF, FM and VAT in both intervention groups were significantly different (*P* < 0.05) from the CON group, but there were no significant differences between two interventions.

### Cardiorespiratory fitness and blood pressure

**Fig. 2:**
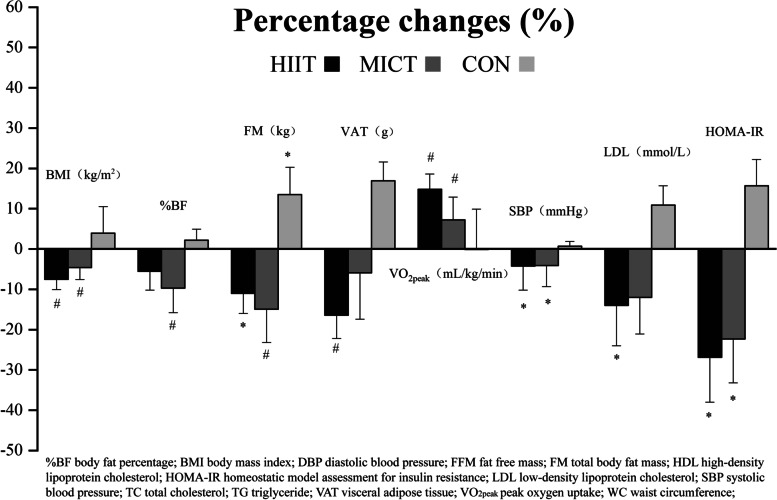
Association between changes in visceral adipose tissue and peak oxygen uptake

After 12-week interventions, both HIIT and MICT program significantly (*P* < 0.01) increased $\dot{\mathrm{V}}{\mathrm{O}}_{2\mathrm{peak}}$ (ES = 0.98 and 0.47, respectively) and decreased SBP (*P* < 0.05, ES = 0.37 and 0.35, respectively), however, the increase of $\dot{\mathrm{V}}{\mathrm{O}}_{2\mathrm{peak}}$ in HIIT group was significantly higher than that in MICT group (6.0 ± 1.5 mL/kg/min vs. 3.8 ± 1.5 mL/kg/min, *P* < 0.01). The DBP of the intervention groups did not change significantly, but it was significantly lower than that of the CON group (*P* < 0.05). In addition, the $\dot{\mathrm{V}}{\mathrm{O}}_{2\mathrm{peak}}$ were negatively and highly correlated (R^2^ = 0.67, *P* < 0.01) in all HIIT, MICT and CON groups with VAT (Fig. 2).

### Cardiometabolic markers

Table 3 showed that both the HIIT group (*P* < 0.05, ES = 0.34) and MICT group (*P* < 0.05, ES = 0.34) experienced significant positive modifications for HOMA-IR compared to baseline value, and there was no significant difference between the interventions. Moreover, the LDL of HIIT group was significantly decreased (*P* < 0.05, ES = 0.38). After training, there was no significant change in other cardiometabolic markers.

## Discussion

The main objective of this study was to compare the effects of school-based HIIT versus MICT on health-related parameters in obese boys. The present results demonstrated that school-based interventions as a main form of running were effective in improving body composition and cardiorespiratory fitness of obese boys. Moreover, there is no significant difference between HIIT and MICT.

**Fig. 3:**
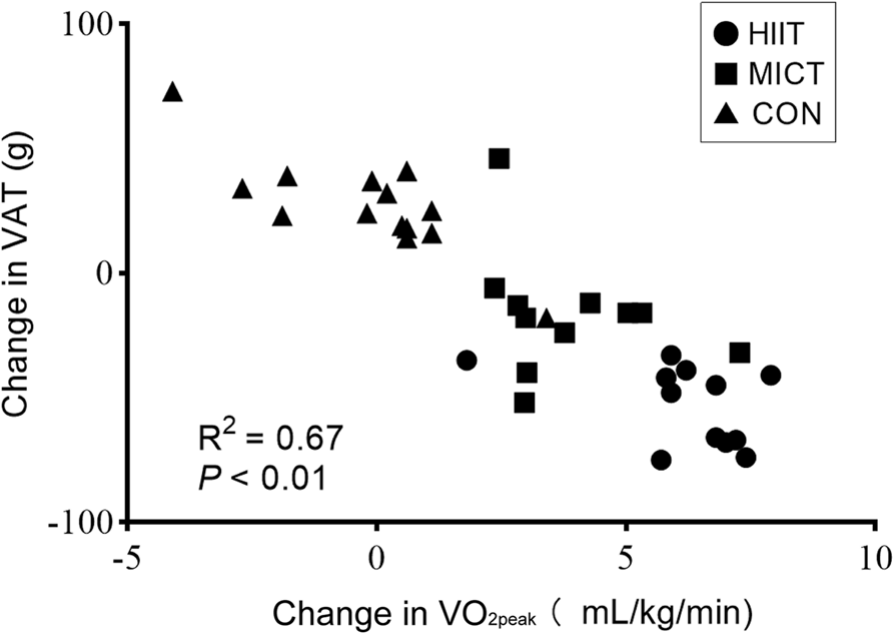
Exercise-induced change in BMI, %BF, FM, VAT, $\dot{\mathrm{V}}{\mathrm{O}}_{2\mathrm{peak}}$, SBP, LDL and HOMA-IR in the three groups after the intervention. *Note*: Values are mean ± SD, ^*^ *P* < 0.05, ^#^ *P* < 0.01

Physical activity increases energy expenditure, which has been proved to be one of the most sustainable and beneficial approaches to prevent and/or counteract childhood obesity [29]. The present study demonstrated that both HIIT and MICT interventions for 12-week effectively improved body composition, reduced BMI and FM in obese boys. Although there were no significant differences in reducing BMI and FM between two training interventions observed in the present study, an interesting finding was that HIIT seems to have a more pronounced tendency to reduce visceral fat (VAT decreases ~ 17%), while MICT tends to decrease the body fat (%BF decreased ~ 10%), Fig. 3 showed the percentage change. Obesity is an abnormal accumulation or excess of fat that can have adverse health effects, especially VAT, which is strongly associated with cardiovascular disease risk [30]. Previously, Racil et al. [21] compared the effect of moderate-intensity interval training (MIIT) vs HIIT (3 sessions a week for 12 weeks) on obese adolescent girls and found that reduced BMI and central adiposity (measured by WC) were more significant following HIIT than MIIT, moreover, the authors considered that HIIT may activates the preferential oxidation of VAT. Recent studies suggested that the mobilization of IL-6 and irisin, myokines secreted after high-intensity exercise but not MICT, may be the potential reasons for reducing VAT [31, 32]. Excess post-exercise metabolism may also be responsible for HIIT induced reduction in central adiposity, since HIIT seems to increase the obese children’s resting metabolic rate (RMR) [7], however, this inference is controversial, no significant changes were observed after three weeks HIIT intervention in RMR [33]. Islam et al. [34] demonstrated that acute exercise augmented post-exercise oxygen consumption (EPOC) and fat utilization, which showed an intensity-dependent manner, with a greater impact following SIT (4 × 30 s “all-out” sprints with 4 min of rest) than MICT (30 min running at 65% $\dot{\mathrm{V}}{\mathrm{O}}_{2\max }$). However, considering the higher intensity of SIT, whether HIIT has a similar effect needs to be further studied. Lee et al. [35] reported that HIIT showed more favorable effect than MICT for decreasing post-prandial insulin and triglyceride levels, and increasing fat oxidation in the next morning.

Overweight and obese children have been shown to possess lower CRF than normal weight children, which increases the risk of developing cardiovascular diseases [36]. Even a slight increase in CRF can significantly reduce the risk of cardiovascular diseases and premature mortality [37]. The present study confirmed that CRF (i.e., $\dot{\mathrm{V}}{\mathrm{O}}_{2\mathrm{peak}}$) in obese boys was significantly increased following 12-week school-based training interventions (Table 3 and Fig. 3), which was increased more in HIIT (+ 15%) than MICT (+ 9%). Despite many evidences from laboratory were consistent with the present study [7, 38, 39]. Similar results were also found in a recent study from Silva [40] and Bogataj [41], school-based HIIT intervention can significantly improve the CRF of overweight/obese students. In addition to the different measurement methods (6MWT and YYIRT1) from this study, one is based on school PE class [40], while the subjects of another study are overweight girls [41]. In the present study, we showed that in a real-world scenario, HIIT can use less time (11 min vs. 30 min) to achieved similar effects with MICT in improving body composition and CRF.

Fasting insulin and HOMA-IR are indicators of insulin resistance. After training, fasting insulin decreased in intervention groups but were not statistically significant (*P* = 0.06). However, HOMA-IR was significantly decreased. To date, few studies have shown improvements in glucose homeostasis of obese children following HIIT [26, 27, 39], others demonstrated that HIIT has no effect on blood glucose and insulin [38]. However, due to the relatively few studies and inconsistent results, it is difficult to confidently make conclusions on the impact of HIIT compared to MICT on glucose regulation. Well-powered randomized controlled trials should be conducted to address these gaps in the future.

HIIT significantly decreased LDL levels is another meaningful finding of this study. Decreases in LDL is important as this decreases the risk of cardiovascular diseases [42]. This is consistent with the results of previous HIIT intervention studies on overweight/obese young men [43], while others showed no significant effect [27, 38]. The discrepancies could be related to differences in subject characteristics (ethnicity, diet, and previous physical activity), training intensity and duration.

Hypertension causes organ damage in obese children, such as left ventricular hypertrophy and endothelial dysfunction [44]. In the present study, we found that 12-week HIIT intervention could significantly decrease SBP (~ 4%). Overweight/obese children have a higher prevalence of hypertension. Development of obesity increases the likelihood of hypertension in childhood leading to future hypertension [45]. Although the subjects in this study were not hypertensive, but they were partly prehypertensive (SBP between 120 ~ 129 mmHg and/or DBP between 80 ~ 89 mmHg). Evidence demonstrated that above 4 mmHg SBP reduction which is expected to decrease CVD mortality by 5 ~ 20% [46]. Considering that blood pressure is closely related to cardiovascular health, HIIT has significance in reducing SBP in obese children. In addition, WC is positively associated with SBP in children with obesity [47], which consistent with the results observed in the present study, both WC (~ 6%) and SBP (~ 4%) decreased in HIIT group. Whether reductions in WC and VAT might be another reason for SBP decline remains to be investigated.

In the present study, the total required weekly training time for HIIT was 33 min (11 min/session × 3 sessions/week), which was much more efficient in comparison with MICT (30 min/session × 3 sessions/week). With shorter exercise duration and similar/better positive improvement, our data confirmed that HIIT could be applied as a more attractive and convenient exercise regimen for obese children. For school-age children, shorter duration time means better exercise adherence and wider application in schools [41, 48, 49].

The strength of this study was the school-based outdoor HIIT running protocol, which requires the least equipment. The ability to use 20 m-SRT and maximal aerobic speed (% MAS) to evaluate training intensity also contributes to the design and wider application of on-field running programs. The present study also has limitations. First, the number of subjects studied is relatively small, and some subjects refused to join the study at the beginning, which may lead to selection bias in the results of the study. In the future, larger sample size of school-based studies is needed to verify the intervention role of HIIT in children and adolescents. Second, only boys were included in this study, which could not better examine the gender differences in the effect of exercise intervention. Quality of the study could be further improved if daily physical activity level of each participant was recorded by a diary or questionnaire with accelerometer. Furthermore, participants’ running intensity was difficult to measure and monitor with training on the track. The use of MAS to evaluated the running intensities in both groups may have resulted in heterogenous response to the training [50]. The maturity of boys during the intervention may also have a certain impact on the results of the study. Exercise prescription based on heart rate, RPE, lactate and ventilation threshold may be a better choice.

## Conclusions

In conclusion, the present study shows that 12-week school-based running HIIT protocol was highly effective in increasing cardiorespiratory fitness when compared with MICT, and have a similar effect on improving body composition of obese boys. In addition, HIIT also effectively reduced visceral adipose tissue, which is more time-efficient than MICT. In recent, there are some school-based studies with large sample size [17, 51], and the role of health indicators for obese children should be further observed in the future.

## Data Availability

The datasets used or analyzed during the current study are available from the corresponding author on reasonable request.

## Abbreviations

20-mSRT: 20 Meters shuttle run test
%BF: Body fat percentage
BMI: Body mass index
CRF: Cardiorespiratory fitness
DBP: Diastolic blood pressure
ES: Effect size
FFM: Fat free mass
FM: Total body fat mass
HDL: High-density lipoprotein cholesterol
HIIT: High-intensity interval training
HOMA-IR: Homeostatic model assessment for insulin resistance
LDL: Low-density lipoprotein cholesterol
MAS: Maximal aerobic speed
MICT: Moderate-intensity continuous training
SBP: Systolic blood pressure
TC: Total cholesterol
TG: Triglyceride
VAT: Visceral adipose tissue
$\dot{\mathrm{V}}{\mathrm{O}}_{2\mathrm{peak}}$: Peak oxygen uptake
WC: Waist circumference;
